# Reninoma: An Uncommon Cause of Renin-Mediated Hypertension

**DOI:** 10.3389/fped.2014.00089

**Published:** 2014-08-15

**Authors:** Peter Trnka, Luisa Orellana, Mark Walsh, Louis Pool, Peter Borzi

**Affiliations:** ^1^Department of Paediatrics and Child Health, The University of Queensland, Brisbane, QLD, Australia; ^2^Department of Paediatrics, Mater Children’s Hospital, Brisbane, QLD, Australia; ^3^Department of Medical Imaging, Mater Children’s Hospital, Brisbane, QLD, Australia; ^4^Department of Pathology, Mater Adult Hospital, Brisbane, QLD, Australia; ^5^Department of Paediatric Surgery, Royal Children’s Hospital, Brisbane, QLD, Australia

**Keywords:** hypertension, blood pressure, kidney, renin, reninoma, juxtaglomerular cell tumor

## Abstract

Reninoma (juxtaglomerular cell tumor) is a rare cause of renin-mediated hypertension. Reninomas are usually diagnosed in adolescents and young adults with occasional reports in younger children. Patients typically present with a long history of headaches leading to a diagnosis of severe hypertension that responds well to antihypertensive treatment targeting the renin-angiotensin-aldosterone system. The clue to clinical diagnosis is the presence of hypokalemia and metabolic alkalosis on the first blood sample drawn before any treatment is instituted. Elevated blood levels of renin and aldosterone confirm the clinical suspicion of renin-mediated hypertension. Diagnostic imaging is employed to identify the source of excessive renin production. While renal ultrasound can miss reninoma, contrast CT or magnetic resonance imaging of the kidneys are diagnostic modalities of choice leading to the correct diagnosis. Renal vein renin sampling with lateralization might help to identify the site of excessive renin production. Nephron-sparing surgery is curative with maintenance of normal blood pressure after discontinuation of antihypertensive medications in the majority of patients. In this paper, we present the case of reninoma in an adolescent girl emphasizing clinical presentation, diagnostic evaluation, and medical and surgical treatment of this rare tumor. We also discuss important points in the management of children presenting with renin-mediated hypertension.

## Introduction

Prevalence of hypertension in children is about 2.5–3%, with some studies suggesting an increasing trend, making elevated blood pressure one of the most common conditions in childhood (1). Severe hypertension (usually defined as blood pressure at least 20 mmHg above the 95% percentile for age, sex, and height) requires immediate medical attention with gradual reduction of blood pressure and thorough investigations for the secondary causes of hypertension (2).

Kidney disease is the most common cause of secondary hypertension – one of the reasons why pediatric nephrologists are usually involved in the management of these children. Increased production of renin from the affected kidneys causes blood pressure elevation, leading to the development of renin-mediated hypertension. Renovascular disease [renal artery stenosis (RAS)], renal parenchymal disease (renal dysplasia, scarring, glomerulonephritis), or renin-secreting tumors (Wilms’ tumor, reninoma) are the most common diagnoses. Reninomas are an uncommon but well-described cause of renin-mediated hypertension with about 100 reported cases to date (3).

Reninomas are benign tumors originating from the cells of the juxtaglomerular apparatus. They produce excessive amounts of renin, leading to secondary hyperaldosteronism. As a consequence of this hormonal activation, patients develop severe hypertension, potassium wasting, and hypokalemia.

Due to the non-specific nature of presenting symptoms (headaches and lethargy being the most common), there is usually a delay in diagnosis of hypertension, sometimes by years. Even when hypertension is diagnosed, the identification of reninoma can be challenging. Medical imaging looking for more common causes of renin-mediated hypertension, such as renovascular or renal parenchymal disease, might miss these small tumors. Contrast imaging studies and selective venous renin sampling might be helpful in identifying the source of excessive renin production. Once correctly diagnosed, reninomas are cured by surgical resection with the majority of the patients becoming and remaining normotensive.

In this paper, we describe the case of reninoma in an adolescent girl, who presented with a long history of headaches suggestive of renin-mediated hypertension. We present our approach to diagnostic evaluation of this patient that led us to the correct diagnosis of reninoma, and discuss the medical management and surgical treatment of this rare tumor.

## Case Presentation

A 13-year-old girl was referred to our hospital for treatment and investigations of hypertension. Eighteen months prior to her presentation, she developed headaches that slowly increased in frequency and intensity, eventually occurring daily at the time of presentation. Her other symptoms and signs included a 2-month history of lethargy and intermittent polydipsia and polyuria. There was no history of nausea, vomiting, syncope, or visual disturbance. Blood pressure measurement done by her primary care provider was 200/120.

The patient was in good physical health with the only past medical complaint of infrequent episodes of asthma. Her only medication was over-the-counter analgesia for headaches. The family history was significant for factor V Leiden deficiency in her father and migraines in her mother and two sisters. There were no family members diagnosed with hypertension or cardiovascular disease.

Physical examination on admission to hospital revealed a well-grown teenage girl. Hypertension was confirmed by blood pressure readings of 180/120 in both arms and similar readings in the legs. Pulses were well palpable in all limbs with no radio-femoral delay. Examination of the heart revealed normal heart sounds and no murmurs. There was no organomegaly or masses palpable in the abdomen. Examination of the neck revealed no bruit or thyroid gland enlargement. Fundoscopy was normal without any evidence of hypertensive changes. Neurological examination was normal.

Hypertensive target organ damage evaluation revealed normal magnetic resonance imaging (MRI) of the brain and structurally normal heart. There was normal systolic function and trivial aortic regurgitation by echocardiogram, likely due to the hypertension. Detailed ophthalmologic examination did not reveal any hypertensive changes. As previously mentioned, kidney function was normal and there was no proteinuria or hematuria present.

Basic laboratory investigations showed normal urinalysis with no blood or protein, and normal renal (creatinine 42 μmol/L) and liver function. The biochemical profile on the first blood sample after admission was normal, apart from mild hypokalemia (3.0 mmol/L), mild metabolic alkalosis with pH 7.44, and mildly elevated serum bicarbonate (28 mmol/L). Twenty-four-hour urine collection revealed normal sodium (102 mmol) and potassium (53 mmol) excretion in 1.7 L of urine. Because of the association of long-standing severe hypertension and hypokalemia, our further work-up focused on the causes of renin-mediated hypertension.

Hormonal studies showed normal plasma and urinary catecholamine and cortisol levels, and normal thyroid function. Plasma renin (mass) and aldosterone levels were markedly elevated [1380 mU/L (normal range 3–40 U/L) and 1940 pmol/L (normal range 100–900 pmol/L), respectively].

**Figure 1:**
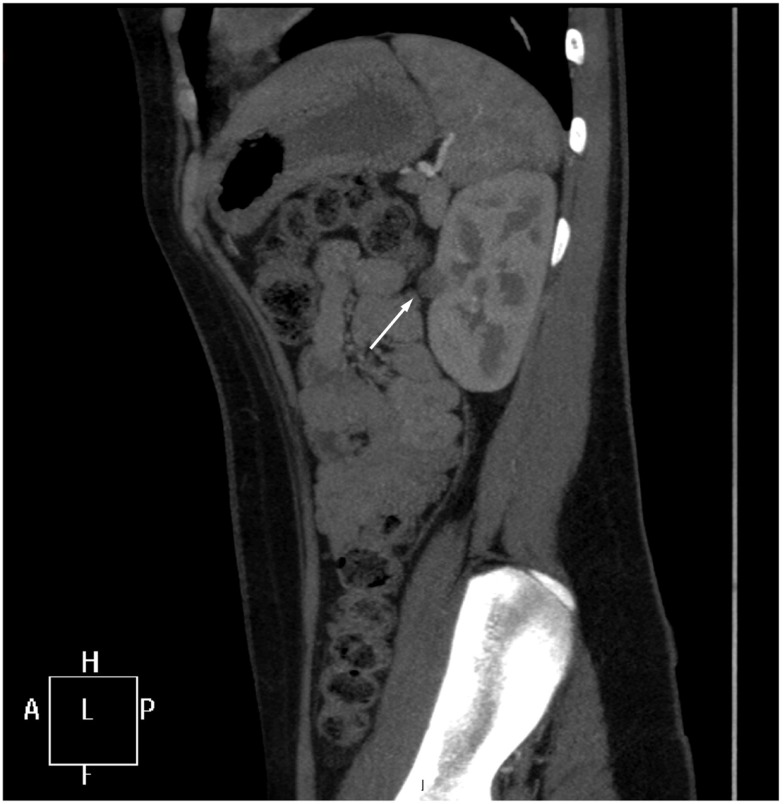
**CT angiogram**. A sagittal reformat through the left kidney shows a rounded 10 mm low-attenuation lesion projecting from the extreme periphery of the anterior renal margin, appearing subcapsular in position (arrow).

**Figure 2:**
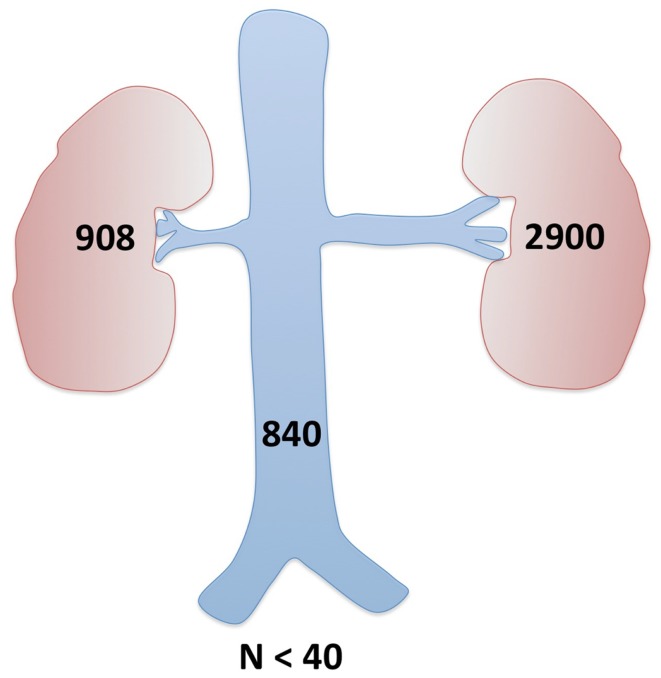
**Renal vein renin sampling with lateralization**. Demonstration of the differential hormone levels between the main renal veins and elevation above systemic hormone levels (all above the upper limit of normal) confirmed the left kidney tumor as a source of excessive renin production.

Further imaging studies were aimed at localizing the source of increased renin production. Abdominal ultrasonography did not reveal any abdominal masses and showed two structurally normal kidneys, with normal renal blood flows (demonstrated by Doppler studies). Computed tomographic angiography (CTA) excluded RAS, but revealed a rounded 10 mm diameter subcapsular mass under the surface of the anterior midpole of the left kidney (Figure 1), suggestive of reninoma. To confirm excessive production of renin from this tumor, renal vein renin sampling with lateralization was performed. The procedure was done under general anesthetic and ultrasonographic guidance. 4Fr catheters were inserted into the proximal vein orifices of the left and right renal vein and sampling for plasma renin mass was undertaken. Plasma renin sample from the left renal vein was significantly higher (2900 mU/L) when compared to the right renal vein (840 mU/L) and the sample from the peripheral vein (908 mU/L), with a L:R ratio of 3.45:1 (Figure 2).

The patient remained clinically well during her stay in the hospital. Hypertension was treated with a combination of calcium channel blocker amlodipine (10 mg/day) and α-blocker prazosin (2 mg three times per day) with normalization of blood pressure (120/80) and resolution of headaches. We did not use medications targeting the renin-angiotensin-aldosterone axis [renin inhibitors, angiotensin converting enzyme inhibitors (ACEI), angiotensin receptor blockers, or aldosterone antagonists] to avoid interference with renin/aldosterone sampling. Hypokalemia was corrected with potassium supplementation.

**Figure 3:**
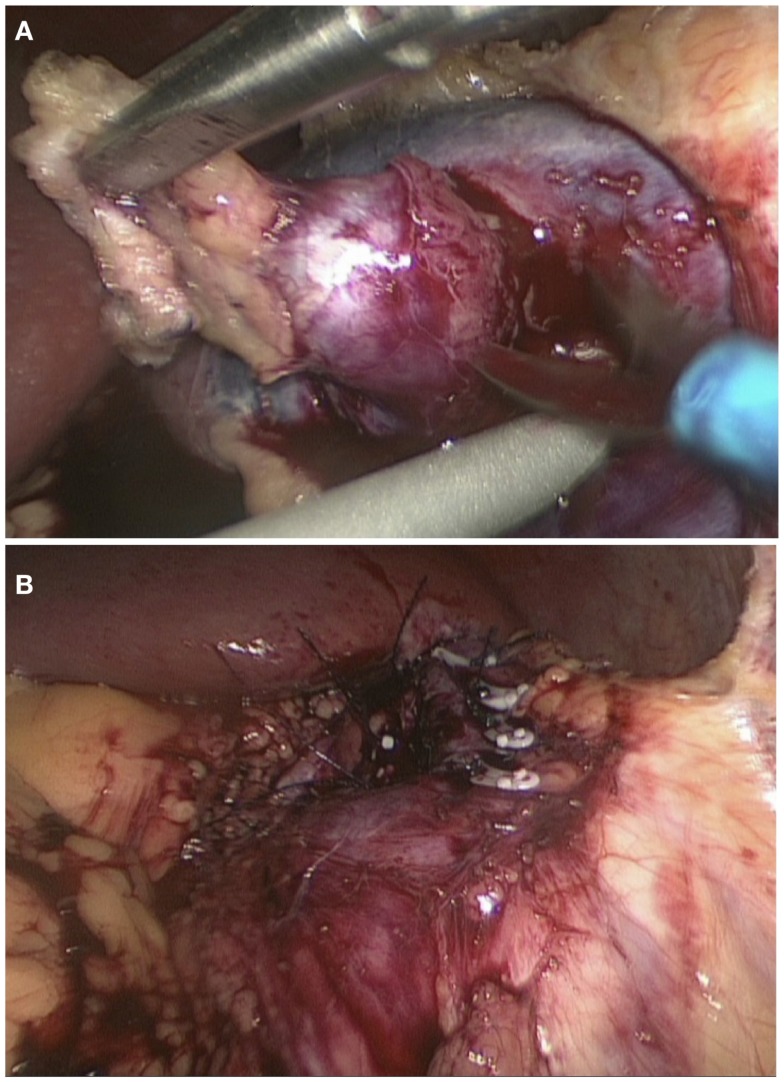
**Laparoscopic wedge resection of reninoma**. Dissection of the tumor with scissors **(A)** and the closure of the defect with interrupted mattress suture **(B)**.

Following the confirmation of the renin-producing tumor in the left kidney, surgical resection of the tumor was performed. The patient was placed in the lateral position with left side up. A laparoscopic transperitoneal approach, with mobilization and reflection of the splenic flexure, allowed exposure of the anterior surface of the left kidney. The vessels were dissected and clamped. The tumor was removed by sharp dissection (Figure 3A). The parenchymal defect was closed with interrupted mattress suture and snugged with a Hem-o-lok (Weck^®^) to secure hemostasis (Figure 3B). The specimen was retrieved inside an endocatch bag as to not contaminate the abdominal wall. Warm ischemic time of the procedure was 18 min. Blood pressure immediately after the surgery was 100/50. Prazosin and potassium supplements were subsequently discontinued, and amlodipine was reduced to 5 mg per day with good control of blood pressure (120/80).

**Figure 4:**
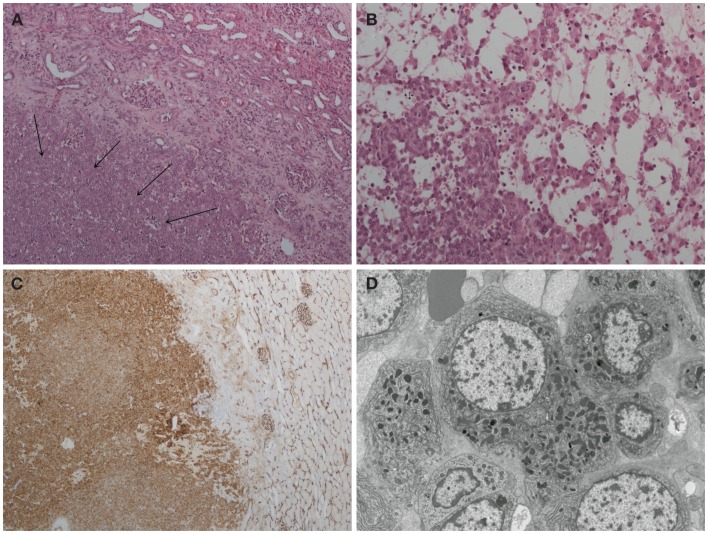
**Histopathology of reninoma**. **(A)** Hematoxylin & eosin, ×200 magnification. The arrows point to juxtaglomerular cell tumor (reninoma). An incomplete fibrous capsule (arrow bases) separates the tumor from normal kidney (the upper right-hand corner). **(B)** Hematoxylin & eosin, ×400 magnification. The tumor comprises a uniform population of round to polyhedral cells with granular, eosinophilic cytoplasm. The growth pattern is solid with edema and single cells. **(C)** CD34 immunoperoxidase stain, ×40 magnification. The tumor cells stain positive for CD34. Adjacent glomerular vasculature acts as an internal positive control. **(D)** Electron microscopy. The tumor cells are characterized by uniform non-enlarged nuclei and a cytoplasm shows numerous pleiomorphic (rounded, polygonal, and rhomboidal) dense deposits, consistent with renin crystals.

Histological examination of the tumor revealed a well-circumscribed lesion measuring 15 mm in diameter with cystic and hemorrhagic changes. The tumor was separated from the normal kidney parenchyma by an incomplete fibrous capsule (Figure 4A). The tumor cells were uniform with round nuclei and eosinophilic granular cytoplasm (Figure 4B). All tumor cells stained positive for CD34 (Figure 4C) but were negative for desmin, CD31, S100, HMB-45, synaptophysin, chromogranin, and WT-1. Constellation of these histological and immunohistochemical features was consistent with the diagnosis of juxtaglomerular cell tumor (reninoma). The diagnosis was confirmed by electron microscopy revealing the presence of rhomboid dense deposits (renin crystals) in the cytoplasm of the tumor cells (Figure 4D). Cytogenetic examination of the tumor revealed normal female karyotype 46, XX without any chromosomal abnormalities.

## Discussion

Reninomas (juxtaglomerular cell tumors) are a very rare cause of renin-mediated hypertension. They originate from the modified smooth muscle cells of the afferent arteriole of the juxtaglomerular apparatus (4). Since the first report by Robertson et al. in 1967 (5), approximately 100 cases of reninoma have been published (3). They are typically diagnosed in adolescents and young adults with occasional cases reported in younger children (6–9). There is a mild female to male predominance with a ratio 1.8:1 (7). Reninomas are benign tumors with only one reported malignant case (10).

Patients with reninoma usually present with a long history of symptoms suggestive of severe hypertension, such as headaches, malaise, or failure to thrive in younger children. Other reported symptoms are nausea, polyuria with polydipsia, and fatigue (11). As symptoms are non-specific, there is often a delay in diagnosis of hypertension (12).

Our patient presented with long-standing headaches, followed by the development of lethargy, and polyuria with polydipsia. Her headaches were initially treated symptomatically due to a positive family history of migraines. Hypertension was diagnosed only after several office visits. Blood pressure measurement performed on presentation, or during subsequent visits to the primary care provider (perhaps as part of a routine screening assessment) could have led to a correct diagnosis earlier.

Similar to our patient, many of the reported cases of reninomas presented with a long-standing history of headaches with a significant delays in diagnosis of hypertension (7, 9, 11, 13). The majority of these patients were children or adolescents.

### Clinical and biochemical consequences of high renin production

Autonomous and persistent secretion of renin by the juxtaglomerular cells of reninoma leads to the activation of angiotensin and stimulation of aldosterone secretion by the adrenal glands. Clinical consequences of this renin-angiotensin-aldosterone system (RAAS) activation are peripheral vasoconstriction and salt retention leading to systemic hypertension. High aldosterone stimulates sodium–potassium exchange by the principal cells of the collecting duct leading to excessive potassium loss in urine, depletion of potassium stores in the body, and stimulation of proton secretion by the intercalated cells of the collecting duct of the kidney (14), with the development of hypokalemia and metabolic alkalosis. These pathophysiologic changes are not specific for reninoma and are usually present in all renin-mediated causes of hypertension. Patients with reninoma, however, tend to be older than children with renovascular or renal parenchymal disease, and usually have no or minimal past medical complaints.

Assessment of urinary excretion of electrolytes is useful but requires 24-h collection of urine. Potassium excretion is commonly within normal daily limits; however, this is inappropriately normal excretion, given the depletion of body potassium stores. Both, the degree of hypokalemia and the amount of potassium excreted in urine, are influenced by the dietary intake of potassium. Hence, a review of the patient’s diet by a dietician is helpful. Our patient presented with hypokalemia and mild metabolic alkalosis. A urine electrolyte excretion study confirmed inappropriately normal potassium excretion (contrary to what we would expect given the low blood level of potassium), suggestive of hyperaldosteronism.

In hypertensive patients presenting with hypokalemia (and sometimes metabolic alkalosis), high aldosterone is the likely cause, guiding the investigations toward renal or adrenal causes of hypertension. Ideally, the measurement of plasma and urine electrolytes is done before any treatment (including intravenous fluid administration) is instituted.

In cases of suspected renin-mediated hypertension, the most physiological choice of antihypertensive treatment is the blockade of RAAS (15). This can be done at multiple levels of the axis by using renin inhibitors (aliskiren), ACEI, angiotensin II receptor blockers (ARB), or aldosterone antagonists (spironolactone, eplerenone). Traditionally, ACEIs and ARBs have been most commonly used in clinical practice. It is important to remember that these medications affect the production and secretion of RAAS hormones and thus alter the blood levels of these hormones. For that reason, we treated our patient’s hypertension with a combination of calcium channel blocker (amlodipine) and α-blocker (prazosin), which allowed us to sample renin and aldosterone without interference. We achieved good blood pressure control with this combination of antihypertensives, and therefore continued with this treatment until the surgical removal of reninoma.

### Hormonal studies for renin-mediated hypertension

The next step in diagnostic evaluation of hypertensive patients presenting with hypokalemia and metabolic alkalosis is the measurement of renin and aldosterone blood levels to confirm renin-mediated hypertension. High renin and high aldosterone combined with a normal renin/aldosterone ratio are suggestive of normal, albeit excessive, secretion of both hormones typical of the renal causes of hypertension (RAS, renal parenchymal disease, or reninoma). A high aldosterone level with a suppressed level of renin point to the adrenal cause (tumor, hyperplasia), where abnormal autonomous hypersecretion of aldosterone suppresses the secretion of renin by the kidneys. The production of renin and aldosterone can be influenced by many factors, including the state of hydration, dietary intake of salts, postural changes, and medications (16). The levels of these hormones can also fluctuate with time, but the elevation above the upper range limit is usually sustained.

We routinely perform three measurements of renin and aldosterone blood levels while avoiding medications targeting RAAS. In our case, both renin and aldosterone blood levels were significantly elevated, with a normal renin/aldosterone ratio confirming the appropriate activation of RAAS, suggestive of renal cause of hypertension.

### Imaging of renin-mediated hypertension

As a particularly rare cause of hypertension, reninoma is seldom strongly considered in the imaging work-up in cases of pediatric hypertension. As characteristically small lesions at presentation (≤10 mm), reninomas are easily overlooked at imaging – imaging typically directed at identifying a vascular cause for renin-mediated hypertension (17).

Plain radiographs are of limited diagnostic value. Occasionally, secondary signs of hypertension such as cardiomegaly and aortic unfolding or cardiogenic or “flash” pulmonary edema will be evident on chest radiography.

The Doppler interrogation of the renal vasculature dominates the ultrasound (US) investigation of hypertension, which detracts from a detailed B-mode evaluation of the kidney substance – whether in search of renal scarring or tumor. Further compounded by a child’s limited tolerance of protracted examinations, in practice US is not a reliable means of evaluating for vascular or neoplastic causes of pediatric hypertension.

Computed tomographic angiography is generally considered the mainstay technique in the imaging evaluation of renin-mediated hypertension in children. Though significant, the radiation burden associated with CT is justified through a number of advantages over other modalities. When compared to MRI, CT is readily accessed and performed, possesses superior spatial and temporal resolution, is well tolerated amongst children, and, notably, is the favored modality amongst interventionalists and surgeons when planning procedures. For patients with diminished renal function when the nephrotoxic effects of iodinated contrast may be amplified, as with gadolinium-based contrast for MRI, specific measures such as pre-hydration may be necessary. CTA has been reported to have 100% sensitivity in diagnosing even small reninomas (11).

Formal renal angiography, though not specifically indicated in cases of reninoma, is often performed in cases of suspected RAS and in cases likely requiring radiological intervention such as balloon angioplasty or pre-operative tumor embolization.

In this case, renal US missed the diagnosis of reninoma. Because of the normal appearance of the kidneys, we suspected a renovascular cause of hypertension. CTA excluded RAS but revealed the small subcapsular tumor (reninoma) of the left kidney as a suspected cause of hypertension. This tumor would also likely have been missed on MRI because of its small size. Conventional angiography, which was briefly contemplated for the patient, would have also missed the tumor (18).

As an excellent means of imaging of the renal vasculature, renal parenchyma, and adrenal glands, we recommended that CTA be considered in all cases of renin-mediated hypertension in children.

### Selective renal vein renin sampling with lateralization

Renin sampling from both renal veins (lateralization) and the peripheral vein helps to localize the site of renin production (4, 13). It has been suggested that renin lateralization ratio (LRR) > 1.5 maximizes the specificity of this investigation (11). Our patient’s LRR was 3.45 favoring the left kidney, confirming that the lesion seen on CT was producing excessive quantity of renin. Following this confirmation, the risks of surgical resection could be justified.

### Surgery for reninoma

Surgical resection of reninoma is curative and restores normal blood pressure in the majority of patients. Occasional cases of persistent hypertension following reninoma resection are thought to be either due to other causes of hypertension, or to cardiovascular changes from long-standing hypertension (12, 19, 20). Whether these patients eventually become normotensive is difficult to predict given no reports on long-term follow-up.

In designing the optimal approach for our patient and her renin-secreting tumor, we appreciated the likely benign nature of this lesion (20). To preserve as much normal functional nephron mass as possible, nephron-sparing surgery in the form of partial nephrectomy, wedge excision, or even tumor enucleation are all possible alternatives to complete nephrectomy for reninomas.

The surgical procedure is done either by open or, more commonly, laparoscopic approach (21). Minimal access surgery ensures better magnification, improved postoperative recovery profile, and better cosmetic results compared to traditional open renal surgery. Complete resection of reninoma with an adequate rim of normal kidney tissue (at least 0.5 cm) is important (22). Warm ischemic times are usually less than 30 min with no cases of acute kidney injury reported in the literature. The surgery is well tolerated and most patients are discharged home within a few days after surgery.

Although both retroperitoneal and transperitoneal minimal access routes have been described (22, 23), we adopted the transperitoneal route in our patient because of a large experience with transperitoneal partial nephrectomy for adult renal tumors in our department. This allowed us to perform a complete, safe, and successful wide excision, within tolerable warm ischemia time of < 30 min.

### Histopathology of reninoma

Histological examination of the excised tumor is essential for confirming the diagnosis of reninoma because other renal tumors can also secrete renin (24). The correct diagnosis is based on the combination of macroscopic appearance of the tumor, light microscopy of the tumor cells, immunohistochemical staining for various cell markers (including renin), and typical ultrastructural finding of intracellular renin deposits (3, 8).

Reninomas are usually small subcapsular tumors, well circumscribed, and mostly encapsulated. Macroscopically, the surface of the tumor is pale or purple with occasional cystic or hemorrhagic areas.

On light microscopy, reninomas are composed of closely packed uniform, round to polyhedral cells with granular, eosinophilic cytoplasm. The nuclei are round to oval with few mitoses. Prominent thick and thin-walled vessels are usually present.

The diagnosis of reninoma is usually confirmed by cytoplasmic positivity for renin. Renin positivity may also be observed in some cases of Wilms’ tumor (WT-1 staining), renal cell carcinoma (RCC), or renal oncocytoma. Most neoplastic cells are also diffusely positive for CD34 (a vascular marker), an additional marker that can differentiate reninoma from RCC (8). Reninomas are negative for S100 protein, HMB-45 (melanoma and angiomyolipoma markers), chromogranin, synaptophysin (neuroendocrine markers), and cytokeratin (a pan-epithelial marker more specific for RCC).

Ultrastructurally, the cells feature rhomboid, renin-specific crystals, similar to those found in normal juxtaglomerular apparatus.

Our patient’s tumor consisted of a uniform population of round to polyhedral cells, with granular, eosinophilic cytoplasm suggestive of juxtaglomerular cells. Immunoperoxidase staining for renin was not available. All tumor cells stained strongly for CD34. Staining for other markers, including cytokeratin, was negative, essentially excluding other kidney tumors. Electron microscopy revealed renin deposits in the cytoplasm of the tumor cells, confirming the diagnosis of reninoma.

Chromosomal abnormalities have been reported in cases of reninoma, with monosomy of chromosome 9, 11, and 15, and polysomy of chromosomes 3, 4, 10 13, 17, and 18 (3). The causative gene(s) are, however, unknown at present. Some studies suggest that the possible oncogene(s) or tumor suppressor gene(s) may be localized on chromosome 4 and 10, or chromosome 9 and 11, respectively (3, 25, 26). No chromosomal abnormalities were detected in our patient.

## Concluding Remarks

Reninomas are a rare but curable cause of secondary hypertension. The diagnosis of reninoma should be considered in patients presenting with severe hypertension, hyperreninemia, hyperaldosteronism, and hypokalemia in whom renovascular and renal parenchymal disease have been excluded. Enhanced imaging modalities and invasive renin sampling are helpful diagnostic tests leading to the correct diagnosis. Antihypertensive treatment with medications targeting RAAS is effective, but can affect the levels of renin and aldosterone if instituted before the collection of the samples for measurement of these hormones. Surgical resection of reninoma (nephron-sparing surgery) is the treatment of choice and leads to normalization of blood pressure.

## Author Contributions

All authors were involved in the care of the patient. Peter Trnka and Luisa Orellana coordinated the diagnostic work-up and medical treatment, Mark Walsh guided imaging diagnostic studies and performed renin vein sampling, Louis Pool conducted the histopathological studies, and Peter Borzi performed the surgical procedure and follow-up. They all contributed to the writing of this manuscript and its revision.

## Conflict of Interest Statement

The authors declare that the research was conducted in the absence of any commercial or financial relationships that could be construed as a potential conflict of interest.
